# Regulation by transcription factors in bacteria: beyond description

**DOI:** 10.1111/j.1574-6976.2008.00145.x

**Published:** 2008-12

**Authors:** Enrique Balleza, Lucia N López-Bojorquez, Agustino Martínez-Antonio, Osbaldo Resendis-Antonio, Irma Lozada-Chávez, Yalbi I Balderas-Martínez, Sergio Encarnación, Julio Collado-Vides

**Affiliations:** 1 Programa de Genómica Computacional, Centro de Ciencias Genómicas, Universidad Nacional Autónoma de MéxicoCuernavaca, Morelos, Mexico; 2 Departamento de Ingeniería Genética, Centro de Investigación y de Estudios Avanzados del Instituto Politécnico Nacional, Unidad IrapuatoMexico; 3 Programa de Genómica Funcional de Procariotes, Centro de Ciencias Genómicas, Universidad Nacional Autónoma de MéxicoCuernavaca, Morelos, Mexico

**Keywords:** regulatory network inference, regulatory network plasticity, chromosome structure, dynamical models of regulatory networks, regulatory network

## Abstract

Transcription is an essential step in gene expression and its understanding has been one of the major interests in molecular and cellular biology. By precisely tuning gene expression, transcriptional regulation determines the molecular machinery for developmental plasticity, homeostasis and adaptation. In this review, we transmit the main ideas or concepts behind regulation by transcription factors and give just enough examples to sustain these main ideas, thus avoiding a classical ennumeration of facts. We review recent concepts and developments: *cis* elements and *trans* regulatory factors, chromosome organization and structure, transcriptional regulatory networks (TRNs) and transcriptomics. We also summarize new important discoveries that will probably affect the direction of research in gene regulation: epigenetics and stochasticity in transcriptional regulation, synthetic circuits and plasticity and evolution of TRNs. Many of the new discoveries in gene regulation are not extensively tested with wetlab approaches. Consequently, we review this broad area in Inference of TRNs and Dynamical Models of TRNs. Finally, we have stepped backwards to trace the origins of these modern concepts, synthesizing their history in a timeline schema.

## Introduction: *cis* elements and *trans* regulatory factors

Transcriptional regulation emerges from the interaction between *trans* factors (Latin for ‘far side of’) that bind to *cis*-regulatory elements (Latin for ‘this side of’) in the context of a particular chromatin/chromosome structure. Taking the doubled-stranded DNA molecule as a reference, *cis* elements are all those DNA regions – encoded in a plasmid or in a chromosome – in the vicinity of a gene. In complement, all the diffusible cellular molecules that are able to bind to the DNA are the *trans* factors. The coactivity of these molecular entities composes the minimal transcriptional regulatory system in all living organisms. In bacterial chromosomes, a transcription unit (TU) is the ordered assembly of the following genetic entities: a regulatory region, a transcription start site, one or more ORFs and a transcription termination site. When a TU comprises more than one ORF, the transcribed mRNA is called polycistronic; otherwise, it is called monocistronic. It is not uncommon for genes to be transcribed by several promoters; thus, TUs overlap. The collection of overlapping TUs constitutes an operon. Historically defined as a polycistronic TU, it has been observed that operons always contain a promoter that transcribes the whole set of genes conforming its TUs. The regulatory region contains *cis* elements such as the promoter – where the RNA polymerase initially binds – and transcription factor-binding sites (TFBS) – where transcription factors (TFs) bind to modulate the binding of the RNA polymerase (Browning & Busby, 2004). In prokaryotes, these regions occupy up to 400 base pairs (bp) (Collado-Vides *et al.*, 1991).

Transcription initiation in bacteria requires proteins known as sigma factors (σ). These factors – with even dozens of different types per genome – are essential for proper promoter recognition by RNA polymerase (Maeda *et al.*, 2000; Helmann, 2002; Paget & Helmann, 2003; Kazmierczak *et al.*, 2005). In bacteria, σ factors are divided into two main phylogenetic families: σ^70^ and σ^54^. The σ^70^ family includes the housekeeping σ that contributes with most of the gene transcription under normal conditions. One subgroup of factors from this family comprises a varying number of proteins known as extracytoplasmatic factors (ECF) activated in response to environmental stress. Usually, every bacterium has one protein member from the σ^54^ family. RNA polymerase associated with a member of this family recognizes promoters that are different from those exclusively recognized when associated with a member of σ^70^. However, there are exceptions where two different σ factors bind to the same promoter (Weber *et al.*, 2005; Wade *et al.*, 2006; Typas *et al.*, 2007). Most σ factors have one anti-σ protein that binds to their σ cognate, inhibiting its action. The σ activity depends on σ/anti-σ ratios and the mechanisms to dissociate σ/anti-σ complexes are diverse (Hughes & Mathee, 1998). Also, there are post-translational mechanisms that modulate the activity of TFs and σ factors such as proteins of transport systems that sequester the factors, releasing them only when special conditions are encountered (Martinez-Antonio & Collado-Vides, 2008).

TFs are classified in several families based on at least two domains, which allow them to function as regulatory switches (Jacob, 1970). One domain functions as a signal sensor by ligand-binding or protein–protein interaction. In many cases, the ligand is a metabolite or a physicochemical signal that conduits the endogenous or environmental information (Ptashne & Gaan, 2002; Martinez-Antonio *et al.*, 2006). The other domain is the responsive element of the switch that directly interacts with a target DNA sequence or TFBS. In bacteria, the helix–turn–helix domain is the most common (Madan Babu & Teichmann, 2003a; Seshasayee *et al.*, 2006). Also, in bacteria, most of these domains are present in one single protein, except for *two-component systems* (Ulrich *et al.*, 2005). Classically, in these systems, when the sensor protein – usually localized in the cell periplasm – senses an exogenous condition, it phosphorylates itself and its cytoplasmic partner, which has a transcriptional regulatory activity (Mascher *et al.*, 2006). These two-component systems work as a unit: evidences from *Escherichia coli* show that 26 of the 29 pairs are encoded in the same operon (Janga *et al.*, 2007a).

In general, negative regulators bind to the promoter, interfering directly with RNA polymerase; in contrast, positive regulators bind to the promoter's upstream region, helping to recruit the polymerase and start transcription (Collado-Vides *et al.*, 1991; Madan Babu & Teichmann, 2003b). TFs usually work as homodimers, tetramers, hexamers and even, in a few cases, as heterodimers (Goulian, 2004). TFs work in concert and a regulatory region can be occupied by several TFs. One of the causes of this crowding of the DNA by TFs in some regulatory regions is the degeneracy of TF–TFBS interaction, i.e. there are different sites that are able to recruit the same TF and different TFs that can recognize similar sites. For example, overlapping regulons like *E. coli*'s SoxS, MarA and Rob arise because of TF–TFBS degeneracy (Martin & Rosner, 2002). The regulatory effect depends on the TF concentration and TF–TFBS affinity: to function, weak sites require high concentrations of TFs; in contrast, strong sites work with a lower amount (Alon, 2007a, b). Also, compared with local TFs that tend to have high-affinity sites, global TFs are less specific, bind to a larger collection of sites and must be expressed at higher levels (Lozada-Chavez *et al.*, 2008; Martínez-Antonio *et al.*, 2008). Furthermore, there are TFs with a dual regulatory role, being activators and repressors at the same time. One simple example are TFs that bind to a single site in the intergenic region between divergently transcribed units, regulating each one of them in a different manner. This is a common theme in sugar catabolism loci where a structural operon is activated, whereas the gene that codes for the TF itself is repressed. An alternative process by which dual regulation works is by the interplay between TF concentration and binding site strength: imagine two TFBSs for the same TF, a weak *negative* site inside a promoter and a strong *positive* site next to it. When the TF concentration is low, the strong positive site recruits the TF and transcription is promoted. As the TF concentration increases, the strong site saturates and the weak site begins to be occupied, thus preventing the union of the polymerase to the promoter. The transcriptional regulator factor for inversion stimulation (Fis) has a dual function over some TUs using the previous strategy (Weinstein-Fischer & Altuvia, 2007).

It is not yet possible to predict the regions of DNA binding from protein structure and experimental mapping is necessary. In general, the number of genes encoding TFs increases with the number of total genes. In particular, in bacterial genomes this increment is proportional to the squared number of genes, suggesting that the increase in genome size is followed by a greater regulatory complexity (Cases *et al.*, 2003; van Nimwegen, 2003; Aravind *et al.*, 2005; Molina & van Nimwegen, 2008). Also, genes in small genomes are relatively more clustered in operons compared with genes in larger genomes (Moreno-Hagelsieb, 2006). However, recent evidences support the idea that the average number of TFBS per regulatory region is independent of genome size (Molina & van Nimwegen, 2008). (Box 1).

**Box 1. Timeline**

**Fig. 1:**
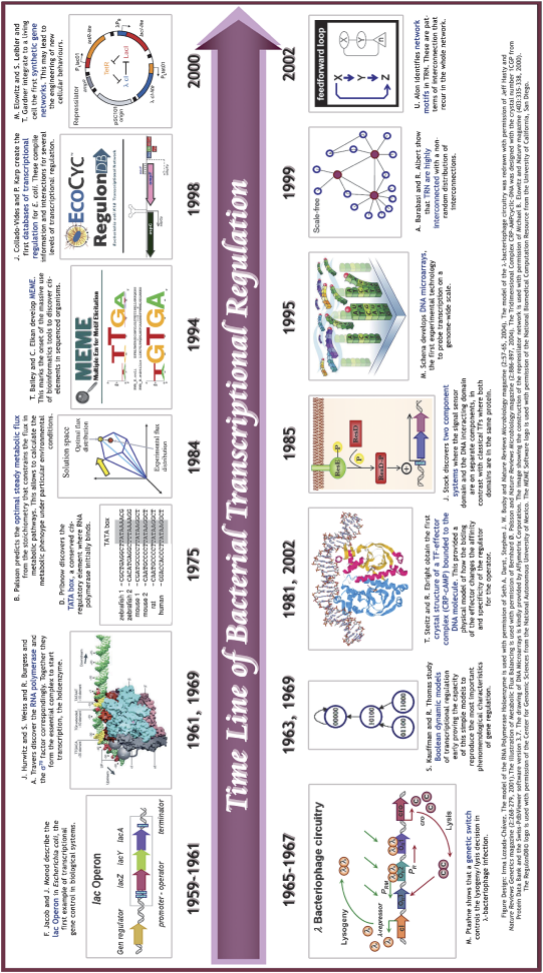
Timeline of bacterial transcription regulation.

Transcription is regulated. This was realized currently with two classical examples: the induction of the *lac* operon (Jacob & Monod, 1959, 1961) and the control of the lytic-lysogenic decision in λ-phage infection (Ptashne, 1965). The circuits controlling these processes are canonical examples that present almost all properties ubiquitous to all gene regulation. It did not take too much time to realize that all cellular functional states, for example cell types, could be codified in a genetic network. This hypothesis gave rise to the first theoretical studies on gene networks that showed that stable genetic patterns indeed arise on very simple models (Kauffman, 1969, 1995; Thomas & D'Ari, 1990). RNA polymerase is essential for transcription (Hurwitz, 1959) and many factors concur to modulate gene expression through the regulation of the binding to DNA of this molecular machine. For example, σ factors and anti-σ factors coordinate the rapid response of many processes in the face of environmental changes and are essential for proper transcription (Burgess *et al.*, 1969; Stevens & Rhoton, 1975). Also, factors can be inherited across multiple cell generations, giving rise to epigenetic phenomena that are not always determined by the DNA sequence (Luger *et al.*, 1997; Bao *et al.*, 2007). Much of the knowledge on transcriptional regulation was *discovered* with many clever experiments. Nonetheless, direct evidence of metabolite–TF–DNA interaction was not available until the first crystallographic structures were obtained (McKay & Steitz, 1981; Weber & Steitz, 1987; Benoff *et al.*, 2002). The continuous accumulation of experimental facts showed that there are generalities on how cells sense the external environment and couple that change to gene regulation: repressible/inducible systems and two-component systems (Savageau, 1974; Stock *et al.*, 1985). However, the disperse increase of these data did not allow a genome-wide analysis. The solution to this problem began with the appearance of comprehensive structured compendia of transcriptional data with authoritative editing (Wingender, 1988; Huerta *et al.*, 1998). Modularity is present in transcriptional regulation, and one of the first evidences was the discovery of the TATA box motif (Pribnow, 1975), a modular element of transcription initiation. The necessity to automatize motif searching was evident, and many computational sequence-searching algorithms emerged out, among them MEME (Bailey & Elkan, 1994). Genes are highly interrelated and this was clear when the pictures of the first – albeit incomplete – transcription networks (Barabasi & Albert, 1999; Guelzim *et al.*, 2002) and high-throughput experiments appeared, i.e. microarrays and ChIP-chip experiments (Schena *et al.*, 1995; DeRisi *et al.*, 1996; Lockhart *et al.*, 1996; Ren *et al.*, 2000). One of the peculiarities of gene networks is that they have an over-representation of *network motifs*, a signature of evolutionary and structural constraints (Milo *et al.*, 2002; Shen-Orr *et al.*, 2002). Transcriptional regulation controls the presence/absence of cellular components, allowing, for example, to metabolize available nutrients. Even though these networks are highly intricate, the metabolic fluxes of bacterial colonies can be predicted (Palsson & Lightfoot, 1984; Palsson *et al.*, 1984). Many technologies and knowledge on gene regulation have converged to synthesize the first gene circuits (Elowitz & Leibler, 2000; Gardner *et al.*, 2000). With the aid of new technological applications, transcription in single cells has been detected, showing that promoter activity is stochastic, producing bursts of proteins when messenger is transcribed (Yu *et al.*, 2006). Furthermore, single-molecule detection in individual cells reports that 90% of the time the LacI repressor is bound unspecifically to DNA, wandering along it until it encounters its operator (Elf *et al.*, 2007) (Fig. 1).

## Chromosome organization and structure

Chromosome compactness might represent a physical constraint to transcription initiation (Willenbrock & Ussery, 2004; Marr *et al.*, 2008). Recent studies suggest that the *E. coli* chromosome is arranged in structural domains with a loop-like conformation, with sizes that range from 10 to 117 kb (Postow *et al.*, 2004; Gitai *et al.*, 2005). The packing of some regions depends on the activity of nucleoid-associated proteins: in bacteria, these are DNA-bending [integration host factor (IHF), HU and Fis] and DNA-bridging proteins [histone-like protein (H-NS)]. The expression of these proteins depends on the growth phase, suggesting a correlation between growth and nucleoid structure (Ali Azam *et al.*, 1999; Luijsterburg *et al.*, 2006; Zimmerman, 2006). In addition, DNA isomerases, DNA chaperones and accessory proteins also regulate DNA access, coiling, bending and packing. Fis recognizes specific TFBSs and in some DNA regions (100–200 bp) clusters of high-affinity Fis sites can be found. However, Fis may also bind nonspecifically to stabilize DNA loops (Skoko *et al.*, 2006). As opposed to Fis-induced bending, H–NS is a condensing agent of the DNA. However, surprisingly, some experiments have shown that it can also have the opposite relaxing effect (Dorman, 2004). It has been suggested that one of the functions of H–NS is to silence horizontally acquired genes, especially those of low GC content (Navarre *et al.*, 2006).

Chromosome size in bacteria ranges from *c*. 0.5 mbp (intracellular pathogens and endosymbionts) to *c*. 9 mbp (free-living bacteria) (Cordero & Hogeweg, 2007; Vinuelas *et al.*, 2007). A chromosome contains from hundreds to thousands of genes that are encoded in both leading and lagging DNA strands. There is a preference for essential and highly expressed genes (such as those for ribosomal proteins) to be localized in the leading strand near the origin of replication (Rocha, 2004). The strategic orientation of these genes has been explained as an advantage for efficient transcription, for example to avoid head-on collisions between the transcription and the replication machinery (Brewer *et al.*, 1992; Mirkin *et al.*, 2006). The G+C content differs among genomes, although regulatory regions have a rich A+T content, an observation related to the access of the transcriptional machinery (Dekhtyar *et al.*, 2008).

## Epigenetics in transcriptional regulation

Inherited stable changes in cell functioning that cannot be explained as the result of mutations or modifications in the DNA sequence are considered as epigenetic (Bird, 2007). Specific molecular mechanisms are responsible for the transmission of particular *acquired characteristics* in a nongenetic manner: biochemical modifications in DNA or DNA-binding proteins can act as epigenetic markers. Bacterial DNA can be methylated in several ways, resulting in *N*^4^-methyl-cytosine (m4C), *N*^6^-methyl-adenine (m6A) and *N*^5^-methyl-cytosine (m5C). Among these three chemical markers, m4C has been clearly related to epigenetic transcriptional regulation besides its relation to other cellular processes (Casadesus & Low, 2006). Epigenetic markers are conserved through bacterial generations thanks to the capacity of methyltransferases to recognize preferentially hemimethylated DNA. This covalent modification can alter the interactions of restriction enzymes or regulatory proteins with DNA by a direct steric effect. In *E. coli*, many genes such as *dnaA* and *trp* can be regulated by Dam methyltransferase (Low *et al.*, 2001). A well-studied specific example of epigenetic inheritance by DNA methylation is the switching of the *pap* operon in the uropathogenic *E. coli*. The operon is regulated by the interplay of two leucine-responsive protein (Lrp)-binding sites. In the repressed state, Lrp binds the proximal site interfering with transcription and Dam methylates the distal site blocking Lrp binding. The operon is derepressed when PapI dimerizes with Lrp. The PapI–Lrp complex has a higher affinity for the distal site, thus freeing the proximal site from Lrp. Dam methylates the proximal site and transcription begins (Hernday *et al.*, 2002). Any of the two states of the *pap* operon is passed on to daughter cells using the methylation signal.

It is not always necessary to have molecular markers for epigenetic inheritance. One commonly unnoticed – and misconceived as a trivial – example is the transmission, to the daughter cells, of the cellular components in the mother's cytoplasm in every cell division cycle. The cytoplasm contains specific factors that *prime* the daughter's transcription in order to recover the transcription state of the mother cell. For example, it is known that low levels of the gratuitous inducer isopropyl β-d-1-thiogalactopyranoside (IPTG) do not derepress the *lac* operon. However, once high IPTG concentrations have induced the transcription of the operon, it is possible to lower the IPTG concentration to noninducing levels and maintain induced a colony previously induced with high IPTG concentrations. This is because daughters of preinduced mothers have a high level of β-galactoside permease in their membranes. This allows them to import, even at low concentrations, IPTG and maintain the *lac* operon derepressed (Casadesus & D'Ari, 2002).

## Transcriptional regulatory networks

The direct influence of TFs over the transcription activity of different *target genes* (TG) is customarily drawn in a network of causal relationships known as a *transcriptional regulatory network* (TRN) (McAdams & Arkin, 1998; Thieffry & Thomas, 1998; Lee *et al.*, 2002a). The network representation unveils the global organization of transcriptional regulation such as its modular and hierarchical structure (Thieffry & Romero, 1999; Ihmels *et al.*, 2002; Segal *et al.*, 2003; Wolf & Arkin, 2003; Barabasi & Oltvai, 2004; Resendis-Antonio *et al.*, 2005; Yu & Gerstein, 2006; Martínez-Antonio *et al.*, 2008) or the fact that on average every TG is controlled by two TFs (Albert, 2005; Aldana *et al.*, 2007). One natural unit in TRNs is the *regulon*: a set of TGs coregulated by the same set of TFs; this concept was originally defined as the group of genes subject to the exclusive regulation of one TF (Maas, 1964). Regulons are divided into *simple* or *complex* if regulated by a single or by multiple TFs, correspondingly. The majority of regulons in bacteria correspond to the last category (Gutierrez-Rios *et al.*, 2003).

The *E. coli* TRN seems to be dominated by probably <10 *global* TFs (Martinez-Antonio & Collado-Vides, 2003). *Local* TFs usually act in concert with global TFs and are also regulated by them, forming a *feedforward loop* motif (Alon, 2007a, b). In *E. coli*, most of the local TFs tend to be encoded in close chromosomal proximity with one of their regulated genes (Janga *et al.*, 2007a). In addition to simple horizontal cotransfer, a biophysical explanation for local TFs and TGs colocalization is that, because the number of local TF molecules is low, they must be close to their regulated target in order to quickly reach their binding site by jumping and sliding along the DNA molecule (Kolesov *et al.*, 2007; Wunderlich & Mirny, 2008). As a rule, global TFs do not regulate each other directly, a phenomenon known as ‘hubs repulsion’ or disassortativity (Song *et al.*, 2006; Takemoto & Oosawa, 2007). As a general observation, the promiscuity of a TF for binding sites diminishes as its local character augments (Lozada-Chavez *et al.*, 2008), and global and local regulators tend to coordinate jointly a general and a particular condition (Balaji *et al.*, 2007; Janga *et al.*, 2007b). Global TFs and some recently duplicated TF pairs can coregulate some TUs, forming a network motif named *bifan* (Shen-Orr *et al.*, 2002). In fact, this motif is a particular class of the complex regulons coordinated by only two TFs. *Escherichia coli*, for instance, has regulons with as many as four to six TFs mutually affecting expression of their TGs. The transcriptional response concentrating regulatory changes – triggered by environmental signals – is partitioned by global TFs as well as by sigma promoter subsets. For example, this is evident when considering *E. coli*'s σ interactions, giving a very clear separation of gene subsets participating coordinately in heat shock, σ^32^ (Nonaka *et al.*, 2006), stress response σ^E^ (Johansen *et al.*, 2006), and stationary-phase σ^S^ (Typas *et al.*, 2007), etc.

Local regulators and nucleoid-associated factors (many of them global TFs) affect the transcription rate of TGs in drastically distinct ways. Evidence shows that nucleoid-associated TFs and DNA-supercoiling induce continuous changes in the transcription rate, whereas local TFs induce discrete changes (i.e. On/Off transcription states). These two aspects have been compared with the analog and digital components of electronic devices (Blot *et al.*, 2006; Marr *et al.*, 2008).

## Plasticity and evolution of TRN

Thanks to the availability of hundreds of sequenced bacterial genomes, one can consider the following evolutionary question: in bacteria, to what extent are TRNs conserved? Recent studies show that TFs evolve much faster than their TGs, suggesting that TRNs in bacteria are highly flexible and dynamic (Lozada-Chavez *et al.*, 2006; Madan Babu *et al.*, 2006). Several reports that analyze different components of TRNs strongly support their plasticity. For example, multiple evidences show that nonorthologous TFs control equivalent pathways, for example the nonorthologous NagC, NagR and NagQ regulate the utilization of *N*-acetylglucosamine and chitin in various groups of proteobacteria (Meibom *et al.*, 2004; Yang *et al.*, 2006). In contrast and to a lesser extent, orthologous regulators may control distinct pathways in different species, for example the orthologous Fur (*Alpha-, Beta-, Gammaproteobacteria*, bacilli and cyanobacteria) and Mur (alphaproteobacterial rhizobial species *Rhizobium leguminosarum* and *Sinorhizobium meliloti*) regulate iron homeostasis and manganese uptake, respectively (Rodionov *et al.*, 2006). Also, even global TFs do not necessarily regulate similar metabolic responses in different organisms (Friedberg *et al.*, 2001; Suh *et al.*, 2002; Derouaux *et al.*, 2004; Moreno-Campuzano *et al.*, 2006). Likewise, as phylogenetic distances decrease, TFBS conservation increases (Makarova *et al.*, 2001; Mazon *et al.*, 2004). However, there are some exceptions to this rule: TFBSs of BirA (regulation of biotin biosynthesis) are highly conserved in Bacteria and Archaea (Rodionov *et al.*, 2002), while TFBSs of ArgR/AhrC (control of arginine regulon) and NrdR (ribonucleotide reductase regulon) are strongly conserved in Bacteria (Makarova *et al.*, 2001; Rodionov & Gelfand, 2005). This suggests that biotin, arginine and ribonucleotide reductase regulatory sites may be ancient. In addition, bacterial species that live in ever changing environments have a tendency to increase the number of encoded stress-responsive TFs and σ ECF; this may be a simple effect of a larger number of regulators encoded in larger genomes (Helmann, 2002). Finally, studies in *E. coli* show that some parts of its TRN are more conserved if they are involved in basic processes (Cosentino Lagomarsino *et al.*, 2007; Salgado *et al.*, 2007).

Several evolutionary processes, such as *duplication* and *horizontal gene transfer* (HGT), must be studied to understand TRN flexibility. For example, loss and duplication of TFs and TFBS may result in regulon expansion, shrinkage, fusions, fissions and even creation and destruction. It is possible to see the contribution of gene duplication at all levels of TRNs (Teichmann & Babu, 2004), although it seems to be more frequent at the bottom layers (Cosentino Lagomarsino *et al.*, 2007; Lozada-Chavez *et al.*, 2008). There are coordinated TF–TG duplications in bacterial TRN. These events account for 38% of the regulatory interactions in *E. coli*'s TRN and 45% in *S. cerevisiae*'s TRN (Teichmann & Babu, 2004; Zhang *et al.*, 2005). The percentages were obtained considering only paralogy within each species; this can mask a convergent evolution within paralogs. For *E. coli*, the previous percentage contrast with the 8% obtained when HGT events are eliminated from the regulatory interactions arose within the *E. coli* lineage (Price *et al.*, 2008).

Although most TFs have paralogs, they seem to have arisen by HGT rather than by gene duplication within the *E. coli* lineage (Price *et al.*, 2008). Moreover, it seems that, in horizontal transfer events, local regulators flow more easily within near phylogenetic distances than global regulators (Lercher & Pal, 2008; Price *et al.*, 2008). Therefore, global regulators are gained and lost more slowly and are even prone to undergoing a slower sequence evolution than other regulators within a bacterial lineage (Rajewsky *et al.*, 2002; Price *et al.*, 2008). This fact does not ensure the maintenance of their global functional role (Friedberg *et al.*, 2001) because the property of global regulation depends on several evolutionary forces and on TF's particular molecular properties (Lozada-Chavez *et al.*, 2008). In addition, genes recently transferred have low expression levels; probably this is a sign of slow but steady integration of transferred genes into the existing regulatory circuits (Taoka *et al.*, 2004; Price *et al.*, 2008). In *E. coli*, the evolutionary rate of TFBSs of horizontal transferred TGs is fast but gradually decelerates with the age of horizontal transfer (Lercher & Pal, 2008). These facts show that TFs and their TFBSs can evolve largely independently, allowing genes to join or leave regulons and allowing regulatory regions to increase their complexity by augmenting the quantity and type of *cis*-regulatory interactions. HGT, complex gene duplication events and an accelerated sequence divergence may mask the discovery of orthologs, making comparative studies of TRN a particularly difficult task; see Box 2.

**Box 2. Inference of TRNs**

Before any biological question about TRNs can be asked, the technical problem of obtaining a reliable network must be solved. There are essentially three methodologically different ways of doing this: (i) by the compilation of different facts reported in research articles whose main interest could have not been to obtain a network, (ii) by ChIP-chip or ChIP-Seq and (iii) by computational methods with DNA sequences, microarrays or scientific articles as input data. We provide a short description of each one of these methods.

**Databases of compiled isolated experiments**

Interactions derived from the literature are the standard to validate any computational or high-throughput experimental inference (Jacques *et al.*, 2005; Munch *et al.*, 2005; Baumbach *et al.*, 2006; Kazakov *et al.*, 2007; Gama-Castro *et al.*, 2008; Sierro *et al.*, 2008). However, not all the annotated regulatory interactions are equally well supported by experimental facts, and subtleties arise. The experience in RegulonDB has dictated that evidences of TF–TG interaction must be divided into at least two categories: *strong* and *weak*. Evidence is strong if, for example, it comes from footprinting or EMSA plus change in expression or binding site mutation plus change in expression. An example of weak evidence is: expression change detected in a microarray plus existence of a binding site – for a certain TF – detected ‘by eye’ by the researcher. Because of its nature, ‘weak’ interactions may become ‘strong’ interactions or may disappear depending on new evidence.

**ChIP-chip and ChIP-Seq**

These high-throughput experimental techniques are designed to locate, *in vivo* and at a genome-wide scale, regions in the DNA where specific proteins bind, in particular TFs. Both techniques start with chromatin immunoprecipitation: cells are treated with a reagent that crosslinks proteins and DNA. Then, cells are lysed and DNA is digested. By immunoprecipitation, all DNA fragments bound to a TF are recovered. The fragments are denatured and amplified. At this point, if ChIP-Seq technology is used, the amplified fragments are sequenced in an ultrahigh-throughput sequencing machine. Detection of binding sites is performed mapping back the sequenced fragments to the genome (Fields, 2007). If ChIP-chip technology is used, fragments are labeled with fluorescent tags to subsequently be hybridized in a special microarray. The microarray may contain only intergenic regions or may be a high-density tiling array (Grainger *et al.*, 2005a; Wade *et al.*, 2005; Cho *et al.*, 2008). All regions that contain a binding site for the TF will have a signal above background in the microarray. The ChIP-chip technique has been used to infer the component of the TRN of *S. cerevisiae* that is under the control of 106 TFs (Lee *et al.*, 2002b; Grainger *et al.*, 2005b, 2006; MacIsaac *et al.*, 2006).

**Computational approaches**

There is a plethora of computational approaches (Albert, 2007; Margolin & Califano, 2007). Here, we enlist some of the core ideas/techniques behind many inference algorithms and give some of the most representative examples in the literature. It is worth mentioning that all these approaches have a high false-positive rate; they are sometimes unable to discern between direct and indirect regulations and some of them do not detect regulatory feedback loops. However, their informative guidance must not be underestimated. For example, consider the detection of regulatory candidates for some arbitrary gene in *E. coli*. Without any type of previous information, the candidates would be *c*. 4500 genes, i.e. every gene in the *E. coli*'s genome. Using for example Mutual information, the set would be reduced to a dozen of putative regulators – a tractable set size.

**Bayesian networks**

A Bayesian approach solves the following problem: given a set of genes and their expression patterns, find the network that explains the observed patterns with the maximum of probabilities (Pearl, 1988; Heckerman, 1999; Neapolitan, 2003). To discriminate among the different possible networks, a score function – known as the Bayesian–Dirichlet metric – is evaluated. This inference method has been applied to propose a TRN in *S. cerevisiae* (Segal *et al.*, 2003).

**Mutual information**

This is the most general way to detect dependence between two variables. The method is used to estimate, from a group of genes and their expression patterns, whether there exists dependence between all possible pairs. An interaction between a pair is proposed if their mutual information is significantly different from that of the same pair but with the expression patterns randomized (Steuer *et al.*, 2002). This is the core idea behind the inference of the TRN of *E. coli* (Faith *et al.*, 2007).

**Discovery of TF-binding sites**

From a collection of TFBSs for a specific TF, it is possible to obtain an estimate of the binding energy between the TF and any arbitrary site. To construct a network, the estimated binding energy between the TF and every possible site in intergenic regions is obtained. The sites with the highest binding energies are proposed as targets, thus inferring an interaction between the TF and the gene in the surroundings of the binding site (McCue *et al.*, 2002; Aerts *et al.*, 2003; Tompa *et al.*, 2005; Chang *et al.*, 2006; Rodionov, 2007; van Nimwegen, 2007).

**Orthology-based algorithms**

From a model organism, where some regulatory interactions are known, the evolutionary and functional relationships between the components of transcriptional regulation can be studied using phylogenetic trees or bidirectional best blast hits, BBHs. With these tools, a search for orthologous counterparts of TF–TG pairs in the model organism is performed in closely related species. When orthologous pairs are found, new regulatory interactions are proposed (Yu *et al.*, 2004). This has been used to show that networks of transcriptional regulation are highly evolvable in Bacteria (Lozada-Chavez *et al.*, 2006; Madan Babu *et al.*, 2006; Price *et al.*, 2007). Some experimental works have supported the orthology predictions and their regulatory extrapolation based on this approach. This is the case of the Lrp regulon within the *E. coli* lineage (Lintner *et al.*, 2008) and of the σ^B^ regulon in Gram-positive bacteria (van Schaik *et al.*, 2007).

**Natural language processing**

First, a lexicon (e.g. a set of gene names) related to transcriptional regulation is compiled. These nouns are concatenated with verbs – to regulate, to inhibit, to promote, etc. – to, depending on certain grammatical rules, discover regulatory interactions in a collection of related scientific articles. Using this method, from 200 000 *E. coli*'s article abstracts, it was possible to recover 395 regulatory interactions with 85% accuracy (Saric *et al.*, 2006).

The regulatory network of *E. coli* can be perturbed globally, rewiring it to a great extent; this might be a consequence of the inherent plasticity of TRNs. For example, Isalan *et al.* (2008) reconnect some global and local regulators and σ factors also by transforming wild-type strains with constructs of almost all possible combinations of these genes with their different promoters. They rewire the network in 600 different ways, every time adding up to five new interactions. Remarkably, in a wild-type genome background, bacterial colonies are viable in 95% of the cases. Another example of network perturbation in a wild-type background shows that mutations in the housekeeping σ factor induce global rewiring (Alper & Stephanopoulos, 2007). The authors show how this rewiring more efficiently solves several problems of metabolic optimization thanks to the interplay of many changes in gene expression that make possible the exploration of complex phenotypes. These results must be confronted with metabolic networks where enzymes have great specificity for their substrates and many catabolic and anabolic pathways are highly conserved. In this respect, metabolic networks appear to be stiff; in contrast, TRN seem to be loose. TFs bind to a broad spectrum of binding sites with different affinity and change targets widely among species. In the light of the previous facts, the rapid adaptation of bacterial organisms to almost every niche on earth is greatly explained thanks to the plasticity of transcriptional regulation.

## Stochasticity in transcriptional regulation

In transcription, all the time TFs are binding to or unbinding from different sequences in the DNA. The greater the affinity, the greater the time they remain bound. If the sequence is regulatory, there is a likelihood that the rest of the transcription machinery assemblies begin transcription before the TF tears off from DNA by thermal fluctuations. In this picture, there is no natural threshold in affinity above which TFs undoubtedly induce transcription. In general, there are a variety of binding sites and for every one of them a TF will have a different affinity, inducing, with some probability, transcription. When promoters are strong and TFs abound, transcription is certain and has a well-defined rate (Elowitz *et al.*, 2002). However, when promoter strength is weak or TF numbers oscillate around the dozens, stochastic fluctuations in the mean TF numbers are very large and transcription becomes ‘noisy’.

In transcription, variability in the number of messages arises from two sources of noise: one *intrinsic* and the other *extrinsic*. In a hypothetical cell with two identical genes, intrinsic noise would cause differences in their number of transcripts. This effect is analogous to the tossing of two identical coins that do not generate the same sequence of heads and tails. Extrinsic noise originates from the cell-to-cell variation of cellular components, for example the exact number of polymerase molecules. Elowitz *et al.* (2002) measured the individual contribution of the two components of noise by the ingenious construct of two fluorescent proteins of different colors in the same plasmid that were subjected – every one of them separately – to the control of a promoter with the same sequence. Transformed with this construct, individuals of ‘noisy’ strains appear under the microscope with any of the two possible colors (intrinsic noise high). In quiet strains, every individual appears with the same color obtained when combining equal quantities of the two fluorescent proteins (intrinsic noise low). Extrinsic noise is obtained when comparing the fluorescence intensity among cells of the same strain.

One fact with profound consequences in the cell fate decision is the metastable gene expression patterns originating from the random fluctuations of the expression of individual genes. The metastability is attained thanks to TRNs that amplify random fluctuations of gene expression and then sustain stable patterns over biological relevant lapses of time. This causes growing isogenetic colonies of microorganisms to differentiate in subcolonies of specialized ‘cell types’*spontaneously* (Maamar *et al.*, 2007; Suel *et al.*, 2007; Chai *et al.*, 2008). Any single cell from an original isogenetic colony can give rise, in turn, to descendants that differentiate in subcolonies that are in the same proportion as the ones in the original colony.

## Transcriptomics

At present, there are basically two options to probe the transcriptional state of the cell: *microarrays* and *ultra-high-throughput sequencing*. In the first technology, different single-stranded DNA probes are designed and arrayed to monitor the mRNA expression of different genes. These transcriptional products, isolated from a culture sample, are tagged with fluorescent proteins and then hybridized in the microarray against their complementary sequences. The intensity of the fluorescence, in the different locations of the array, gives an estimate of the abundance of the different probed transcripts. Microarray technology has been refined since its first appearance in the mid 1990s when they detected exclusively annotated ORFs (Schena *et al.*, 1995). Today, state-of-the-art microarray technology is represented by *high-density whole-genome tiling arrays*. In this implementation, the arrayed set of probes is richer, containing, for example, DNA probes for both intragenic and intergeneic regions. This improvement allows for the identification of complex transcript structures – such as genes in operons – as well as novel short transcripts – such as small RNAs – that would be missed by previous low-density arrays (Reppas *et al.*, 2006). The raw data generated from microarrays must be transformed in two steps: correction for background noise and normalization. The first transformation attempts to eliminate the contribution from unspecific hybridization; the second transformation intends to make gene intensities from different experiments comparable (Quackenbush, 2002). The widespread use of this technology has led to the appearance of useful databases with collections of hundreds of arrays of different bacterial organisms under diverse experimental conditions (Demeter *et al.*, 2007; Faith *et al.*, 2008; Kanehisa *et al.*, 2008).

There are particular problems that are inherent to microarray technology. For example, prior selection of probes in the arrays biases the possible set of transcripts that can be detected; unspecific hybridization cannot be completely eliminated; the differential efficiency of probes makes it impossible to compare the expression of different genes in the same sample, etc. It appears that the solution to these problems is to use the sheer brute force of massive sequencing with the new ultra-high-throughput sequencing technologies (Bennett *et al.*, 2005; Margulies *et al.*, 2005). The idea is simple: sequence all the transcripts that the cell expresses under a particular condition and then map these sequences back to their corresponding regions on the genome to detect presence or absence (Nagalakshmi *et al.*, 2008). Note that the detection of transcripts is not conditioned on a possibly biased set of probes nor on the resolution of the array. This translates into the possible discovery of new gene products. Also, the effect of unspecific hybridization is not present in the sequencing, and comparison between gene transcript levels is possible because the number of sequenced transcripts is directly counted. At least one study has compared microarray and sequencing technology, showing that data in the latter are highly replicable and that the sequencing technology can detect differentially expressed genes between two samples at a higher positive discovery rate (Marioni *et al.*, 2008).

The processing of transcription data and the rationale behind that same processing is as important as the technology to probe transcription. The traditional data workflow screens for differentially expressed genes; this proceeding has been described, pejoratively, as *fishing expeditions* (Gibson, 2003). This criticism indirectly points to the fact that the community lacks methods to synthesize gene expression data and methods to analyze this synthesis at higher levels of description, for example gene expression data organized coherently in TRN or genes of related function sorted out in functional classes. One way to amend this situation is the use of a clustering method known as Self-Organizing Maps. This clustering reorganizes transcription data in such a way that genes with similar expression levels are contiguously located in a squared lattice, generating an *image* of the state of the transcriptome. Surprisingly, with this reordering, it is possible to sort out different cellular functional states just by seeing the image, a *gestalt* analysis (Guo *et al.*, 2006). Another method of higher level analysis is to take advantage of the decades of molecular knowledge and organize transcriptional data into sets of genes that together perform a cellular process (Subramanian *et al.*, 2005). This *gene set analysis* has a higher statistical power to discriminate changes at the gene set level that would be unnoticed at the single-gene level. (Box 3).

**Box 3. Dynamical models of TRNS**

Dynamical models of TRN present different degrees of granularity that are appropriate to particular aspects of and questions in cell regulation. In deciding the proper model and its coarseness – perhaps the most important step in modeling – all prior available knowledge is important: number of genes, available physicochemical parameters, TF affinities for TFBSs, kinetic parameters, etc. In general, there are three classes of models with particular levels of granularity: *Boolean, stochastic and continuous models* (Smolen *et al.*, 2000; Bower & Bolouri, 2001; Christensen *et al.*, 2007). It is also possible to combine any of them to produce *hybrid models*.

**Boolean models**

The sigmoidal induction/repression of gene expression by different factors is well approximated by step functions with two states: On/Off (Thomas & D'Ari, 1990). Using this simplification, we can define a TRN model: genes with two states (inhibited or induced), interacting through logical rules (ORs, ANDs or Boolean tables in general) in discrete time steps. Model networks with these modest characteristics are Boolean and they present – remarkably – much of the higher order phenomena sustained by gene networks (Kauffman, 1969; Thomas & D'Ari, 1990). To give but one example: only a few gene expression patterns in Boolean models are stable and have a direct correspondence with gene expression patterns of real cell types (Mendoza & Alvarez-Buylla, 1998; Albert & Othmer, 2003; Huang & Ingber, 2007). Also, recent investigations using the Boolean abstraction of real networks provide a first explanation of how – paradoxically – robustness and adaptability coexist in living organisms (Aldana *et al.*, 2007; Balleza *et al.*, 2008; Nykter *et al.*, 2008).

**Stochastic models**

Stochasticity inevitably emerges when molecular components are present at low cellular concentrations (McAdams & Arkin, 1997; Kierzek *et al.*, 2001). This physical phenomenon generates noise in synthetic and natural circuits (Paulsson, 2004; Mettetal *et al.*, 2006), and its consequences over the phenotype are starting to be explored (Suel *et al.*, 2006). For example, noise constitutes the driving force behind differentiation in isogenetic colonies (Colman-Lerner *et al.*, 2005). Biological and theoretical studies have aided to delineate the regulatory mechanism by which the cell handles noise efficiently and effectively to carry out its biological functions (Gardner & Collins, 2000; Orrell & Bolouri, 2004; Raser & O'Shea, 2005). From a theoretical point of view, stochastic models are the most challenging but also the most realistic ones: there is a precise counting of how, through individual chemical reactions, the populations of every chemical species change. The milestone to simulate stochastic processes is the Gillespie algorithm (Gillespie, 1992). Because of their analytical and computational complexity, the present models do not surpass a handful of chemical species. Two immediate problems must be solved to model systems with several dozens of genetic components: the systematic determination of kinetic constants and the efficient computation of thousands of chemical stochastic equations (Kuwahara *et al.*, 2006; Sanchez & Kondev, 2008).

**Continuous models**

Contrary to stochastic models, continuous modeling assumes that the number of components in TRNs is sufficiently high to assume that concentrations are continuous variables. This framework allows taking into account realistic effects such as complex TF interactions, spatial diffusion of molecules and the gradual variation of mRNA expression, to mention just a few (Bower & Bolouri, 2001; de Jong, 2002). The continuous description has been useful for the design of genetic circuits in synthetic biology (Atkinson *et al.*, 2003; Kaern *et al.*, 2003). Remarkably, this sort of approach allows one to understand the biological function of network motifs. For instance, dynamical analyses of feed forward loops show how this circuit controls the slow activation and rapid deactivation of the regulated gene. Also, the analysis of feedback loops evidence their role as the units behind memory (Alon, 2007a, b). As in stochastic models, the continuous approach is useful to quantitatively analyze the dynamics of TRN only when the topology, the regulatory type of the interactions and the kinetic constants are known (Shea & Ackers, 1985; Kobiler *et al.*, 2005). When kinetic constants are not available, plausible values can be used to obtain the possible dynamical responses of TRNs.

**Hybrid models**

Experimental evidence shows that different levels of cell functioning are carried out at different time scales and at different concentrations of their components – seconds and thousands of molecules in metabolism, minutes and hundreds of molecules in transcription. How these levels can be combined to be consistent between them in a single model constitutes an active field of research (Puchalka & Kierzek, 2004; English *et al.*, 2006; Samoilov & Arkin, 2006; Covert *et al.*, 2008). One solution is hybrid models; these combine any of the above approaches to integrating different cues of cell functioning (Bower & Bolouri, 2001). For example, there are hybrid models that take into account the *continuous* character of the concentration of some transcripts and their *abrupt discrete* change in transcription during regulation (Glass & Kauffman, 1973; Thomas & D'Ari, 1990). Another hybrid model is one in which a noise function is added to the *continuous* concentration of transcripts to introduce *stochastic* fluctuations (Ozbudak *et al.*, 2002). Even though these models are more complex than purely discrete ones, they provide a more approximate picture to transcriptional regulation, making it easier, for example, to relate and compare the models with real transcriptome data.

## Synthetic transcriptional regulatory circuits

The previous sections show the detailed knowledge we have accumulated on transcriptional regulation by TFs. The synthesis of TRNs attempts to go from this understanding to a rational transcriptional network design. It aspires to integrate new complex functions into cell behavior; not just the addition of stationary properties such as the constitutive expression of exogenous proteins but the addition of the dynamically controlled expression of complete gene programs. There are several first examples in this direction that show the feasibility of program integration into cell behavior: rational design of memory circuits (Ajo-Franklin *et al.*, 2007), insertion of complete regulated metabolic pathways (Pfleger *et al.*, 2006), toggle switches (Gardner *et al.*, 2000), oscillators (Elowitz & Leibler, 2000) and the creation of new ways of cell–cell communication (Bulter *et al.*, 2004). In all these cases, small gene circuits compute their output based on the external/internal input signal sensed. Promoters controlling the expression of the genes in the circuit are the essential piece to accomplish the required computations, for it is in this element where signals – transduced by TFs – converge and are integrated. The particular importance of promoters has naturally led to an interest in their characterization and synthesis. For example, with respect to their characterization, it has been shown that, more often than not, the activity of different promoters controlled by two regulators is not a simple OR/AND function (Cox *et al.*, 2007; Kaplan *et al.*, 2008). With regard to their synthesis, now we have available complete characterized libraries of synthetic promoters with different strengths; this last fact was verified indirectly by measuring the specific β-galactosidase activity. Remarkably, more than six orders of magnitude in β-galactosidase activity can be covered using different promoters (Mijakovic *et al.*, 2005). It is also possible to create libraries of regulated promoters by combinatorial synthesis (Cox *et al.*, 2007). This consists of the combinatorial ligation of previously created promoter regions, i.e. sequences that correspond to the distal region (upstream the −35 box), to the core region (between the −35 and −10 boxes) and to the proximal region (downstream the −10 box). These regions contain one operator site for any of the following regulators: LacI, AraC, LuxR or TetR. Using this strategy, thousands of promoters with different regulated strengths can be generated.

Supposing complete characterized libraries of different promoters exist, the main challenge in synthetic circuits still remains: to integrate these small networks into the cell environment without killing the cell, for example without overproducing toxic intermediates or causing metabolic bottlenecks that would inhibit growth. The problem is to find the exact promoter strengths with the correct regulatory region to balance and coordinate the expression of multiple genes. One promising solution is to generate a library of networks and then select the best-performing one under a given criterion. This is the same strategy followed in the directed evolution of proteins, where a library of mutant protein sequences is created and then screened for the best variation of the protein. The difference in the library of networks lies in the fact that the mutations are in the noncoding regions that regulate transcription and translation. One example of this approach is the combinatorial synthesis of intergenic regions in operons to tune the translation of polycistronic transcripts (Pfleger *et al.*, 2006). The approach, without a specific design, generates transcripts with slight variations in intergenic regions that change RNAase cleavage sites, ribosomal binding sites sequestering sequences and mRNA secondary structures. With this technique, it was possible to introduce in *E. coli* a heterologous mevalonate biosynthetic pathway by tuning the expression of three genes in an operon. In one last example of combinatorial synthesis, a collection of 125 different networks was produced from these units: five different promoters regulated by three different TFs (LacI, TetR and λ cI) (Guet *et al.*, 2002). Among the networks, it is possible to find positive and negative feedback loops, oscillators and toggle switches. It must be stressed that all these different network functions can be encoded with the same set of genes, the difference residing only in the interaction graph of the constituent genes.

## Concluding remarks: the need for integrative schemes

Even though recent progress to unravel the underlying mechanisms of transcriptional regulation has been spectacular, the community lacks an integrative framework to direct new advances. In this respect, systems biology in bacteria has the challenge to show its promised capabilities of new levels of integration and understanding combining modeling and experiments of the whole network and cell behavior. To achieve this, there are two complementary procedures: bottom-up and top-down schemes (Beer & Tavazoie, 2004; Bonneau *et al.*, 2007). The former traces its origins to the systems sciences, whose essence is to explore the collective phenomena emerging when integrating its building parts (Bruggeman & Westerhoff, 2007). Bottom-up schemes constitute the base to develop mechanistic models that are useful to discern the transcriptional organization by which the cell faces a genetic or an environmental perturbation at a genome scale (Segre *et al.*, 2002; Covert *et al.*, 2004; Resendis-Antonio *et al.*, 2007). On the other hand, top-down procedures require deductive methods, whose main interest is to identify causal interactions between the individual genes measured by high-throughput technologies (Wagner, 2001; de la Fuente *et al.*, 2002). Successful integration of top-down and bottom-up schemes is not a trivial activity; it requires permanent comparison between types of modeling and its experimental verification to reconstruct a coherent explanation of cell activity. The navigation towards progress here depends on how simplified models can capture the essentials to predict, and the fact that biological systems can be engineered in synthetic approaches, even if they are also extremely interconnected.

This review has focused on regulation by TFs. However, there are other layers of cellular regulation that ultimately influence regulation by TFs. This situation creates feedback loops that transmit information from almost any regulatory layer to any other one in order to maintain cellular homeostasis. This is in clear contrast with the isolated picture of TRN, where a cellular hierarchical decision-making structure is emphasized. Thus, a major conceptual challenge is to change our way of thinking about causality in a complex system with an important connectivity and an important amount of circularity, i.e. feedback loops, in the ‘decision’ network of gene regulation at the whole-cell level.

## Statement

Reuse of this article is permitted in accordance with the Creative Commons Deed, Attribution 2.5, which does not permit commercial exploitation.
